# Small Tympanic Membrane Perforations in the Inferior Quadrants Do Not Impact the Manubrium Vibration in Guinea Pigs

**DOI:** 10.1371/journal.pone.0028961

**Published:** 2012-01-04

**Authors:** Xiuling Zhang, Yanhong Dai, Shuyi Zhang, Wandong She, Xiaoping Du, Xiuji Shui

**Affiliations:** 1 Department of Otolaryngology - Head and Neck Surgery, Nanjing University Medical School, Nanjing Drum Tower Hospital, Nanjing, China; 2 Laboratory of Modern Acoustics, Institute of Acoustics, Nanjing University, Nanjing, China; 3 Hough Ear Institute, Oklahoma City, Oklahoma, United States of America; Claremont Colleges, United States of America

## Abstract

**Background:**

It has been believed that location of the perforation has a significant impact on hearing loss. However, recent studies have demonstrated that the perforation sites had no impact on hearing loss. We measured the velocity and pattern of the manubrium vibration in guinea pigs with intact and perforated eardrum using a laser Doppler vibrometer in order to determine the effects of different location perforations on the middle ear transfer functions.

**Methods:**

Two bullas from 2 guinea pigs were used to determine stability of the umbo velocities, and 12 bullas from six guinea pigs to determine the effects of different location perforations on sound transmission. The manubrium velocity was measured at three points on the manubrium in the frequencies of 0.5–8 kHz before and after a perforation was made. The sites of perforations were in anterior-inferior (AI) quadrants of left ears and posterior-inferior (PI) quadrants of right ears.

**Results:**

The manubrium vibration velocity losses were noticed in the perforated ears only below 1.5 kHz. The maximum velocity loss was about 7 dB at 500 Hz with the PI perforation. No significant difference in the velocity loss was found between AI and PI perforations. The average ratio of short process velocity to the umbo velocity was approximately 0.5 at all frequencies. No significant differences were found before and after perforation at all frequencies (*p*>0.05) except 7 kHz (*p* = 0.004) for both AI and PI perforations.

**Conclusions:**

The manubrium vibration velocity losses from eardrum perforation were frequency-dependent and the largest losses occur at low frequencies. Manubrium velocity losses caused by small acute inferior perforations in guinea pigs have no significant impact on middle ear sound transmission at any frequency tested. The manubrium vibration axis may be perpendicular to the manubrium below 8 kHz in guinea pigs.

## Introduction

There is a significant disagreement about the effects of the tympanic membrane (TM) perforation on middle ear sound transmission although it has been studied for over a century [1]–[7]. It has been believed that location of the perforation has a significant impact on hearing loss. For example, posterior quadrant perforations cause more hearing loss than anterior quadrant perforations because of direct exposure of the round window to sound waves in the posterior quadrant perforations [7], [8]. Additionally, perforations at or near manubrium would cause more hearing loss than those far from manubrium but with comparable sizes [8]. However, Voss and her colleagues have recently compared the stapes velocity, middle ear air-space pressures and acoustic impedance of the TM before and after making controlled perforations in cadaver ears. They found that loss of the magnitude of the stapes velocity was not associated with perforation locations but perforation sizes [4]–[6], [9]. Furthermore, some clinical studies have demonstrated that the perforation sites had no impact on hearing loss in those patients with isolated TM perforations without other middle-ear diseases or with acute TM perforations [10], [11]. Therefore, more studies on effects of perforation locations on the middle ear sound transmission are still needed.

It is well known that the primary function of the TM is to convert sound pressure on its outer surface into displacement of the manubrium, and then the sound wave is delivered to the perilymph fluid in the inner ear by ossicular chain motion. Thus, the TM perforation may firstly result in changes in the manubrium vibration. The umbo is used as the best site for transcanal measurement of the manubrium vibration. In addition, measurement of the umbo vibration also provides an indirect measure of stapes vibration if the ossicular chain is intact [12]. Therefore, we used a laser Doppler vibrometer (LDV) system to measure the velocity and pattern of the manubrium vibration in guinea pigs with intact and perforated TM in order to determine the effects of different location perforations on the middle ear transfer functions.

## Materials and Methods

### Animals and bulla harvest

Eight albino guinea pigs (200 to 350 g) were housed in the Animal Center of Nanjing Drum Tower Hospital, Nanjing University Medical School. The Institutional Animal Care and Use Committee of Nanjing Drum Tower Hospital, Nanjing University Medical School, China, approved this study. However, the committee did not give a permit number. In general, the committee together considers a serious of animal experiment projects every year, and performs a result about the animal experiment projects. All animals had a normal pinna reflex and were free from middle ear diseases. They were euthanized by intraperitoneal injection of pentobarbital sodium (40 mg/kg) and the temporal bones were removed immediately. Parts of the bony ear canal wall were removed to optimize access to the TM. In order to keep the TM and middle ear structures moist, the tympanic bulla was surrounded with IVALON® Nasal Packing (First Aid Bangdage Company, New London, USA) soaked with normal saline solution during the dissections and measurements. The whole measurement process was completed within six hours after euthanization.

### Sound stimulation

A speaker with a signal processing board (Agilent 33220A, USA) and an EX-1480 amplifier (Nanjing Electroacoustic Co. Ltd., Nanjing, China) were used as a sound delivery system. The sound pressure level was monitored by an YDG60-18 microphone (Nanjing Electroacoustic Co. Ltd., Nanjing, China) and a DT-805 sound level meter (China Everbest Machinery Industry Co., Ltd., Shenzhen, China). The tip of the sound level meter probe (AAC Acoustic Technology Co., Ltd., Shenzhen, China) and the microphone were placed beside the tympanic bulla, and the speaker was placed 5 cm away from the eardrum.

The stimulus was a harmonic sine signal with frequencies of 0.5, 1, 1.5, 2, 2.5, 3, 3.5, 4, 4.5, 5, 5.5, 6, 7, and 8 kHz, and the maximum sound level was about 90 dB SPL. The sampling frequency was 128 kHz. Each response took approximately one minute to acquire after being averaged six records.

### Measurements with a laser Doppler vibrometer

The velocities were measured with a laser Doppler vibrometer (LDV, Polytec OFV-5000, Germany). The laser beam was directed orthogonally onto the site of interest on the TM without any reflectors. Then the vibration velocity began to be recorded six times after a good signal to noise ratio (>0.8) was obtained.

### Experimental design and statistical analysis

**Figure 1:**
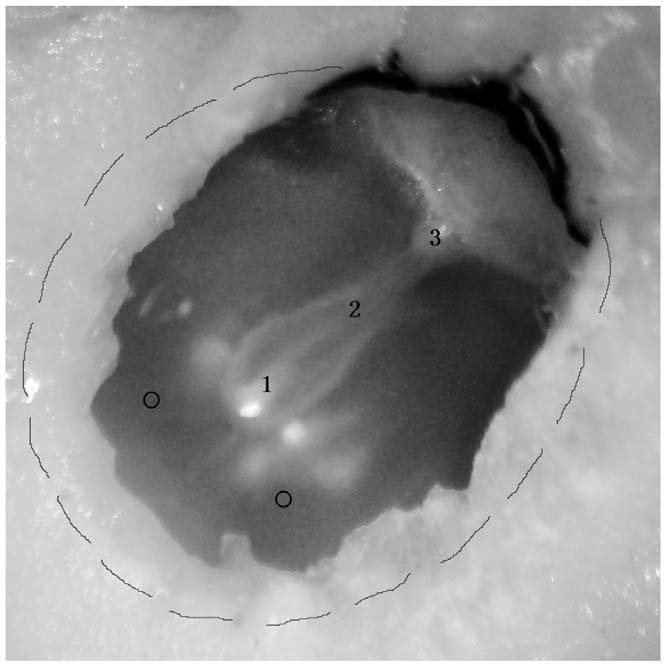
Frequency responses were measured at 3 points on the manubrium of intact and perforated TM. The perforations were made in the anterior-inferior quadrant of left ear and posterior-inferior quadrant of right ear. 1, 2, and 3 on the manubrium represent umbo, middle point of manubrium, and short process of manubrium respectively, and two circlets represent the perforation locations.

Two bullas from 2 guinea pigs were used to determine the stability of the umbo velocities recorded repeatedly over a period of 80 minutes. Twelve bullas from 6 guinea pigs were used to determine the effects of different locations of perforations on sound transmission. The velocity was measured at three points on the manubrium before and after a perforation was made with a 20-gauge needle (1.1 mm in diameter). The locations of the perforations were in anterior-inferior (AI) quadrants of left ears and in posterior-inferior (PI) quadrants of right ears (Fig. 1).

The umbo velocities were averaged in 12 bullas, and standard deviations were calculated for each frequency within a test condition. The significance of the velocity loss resulting from different locations of perforations and the ratio of short process velocity to umbo velocity before and after perforating was statistically analyzed by paired *t*-test.

## Results

### Repeatability

**Figure 2:**
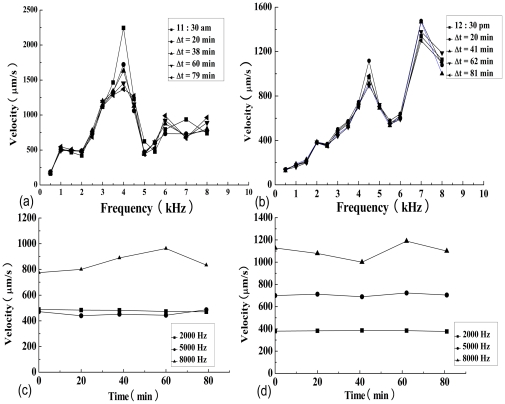
The umbo velocities throughout the frequency range within a time window and the umbo velocities of selected frequencies. These results demonstrate that measurement of the umbo velocity was repeatable over a period of 79- (a) or 83-minutes (b) at selected frequencies (2, 5, 8 kHz).

The velocity responses of the umbo to the harmonic stimulation were measured five times in two bullas from two guinea pigs to examine the stability of the responses. The frequency responses were recorded at the umbo over a period of 79 (Fig. 2a) or 83 (Fig. 2b) minutes. The velocity curves at different time point show the variations of +5 dB to −2 dB compared to the first measurement throughout the frequency range. The time course is illustrated in Figs. 2c and d, which demonstrated stable responses at 2 and 5 kHz over the periods. However, the amplitudes at high frequencies (i.e. 8 kHz) gradually increased (Fig. 2c) or slightly decreased (Fig. 2d) before suddenly dropping after 60 minutes (Figs. 2c and 2d).

### Umbo vibration

**Figure 3:**
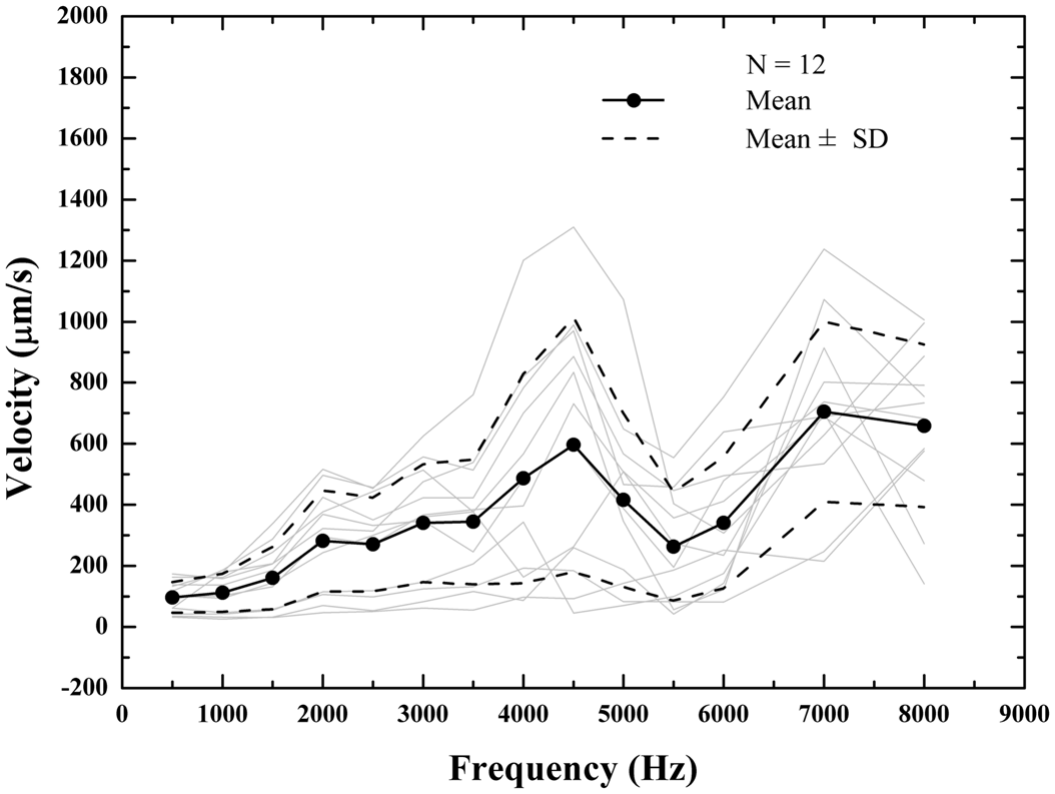
The umbo velocities recorded from 12 bullas with intact tympanic membrane are plotted. The individual measurements were drawn in gray. The thick black solid line represents the mean velocities at each of fourteen frequencies (the filled circles) while the thick black dashed lines represent one standard deviations from the means. The range of velocities was approximately 20 dB throughout the frequency range with two peaks at 2 and 4.5 kHz.

The umbo velocities with means and standard deviations from the intact TMs were shown in Fig. 3. The range of velocities across animals was approximately 20 dB throughout the frequency range. Two peaks in the velocity response were seen at 2 kHz and 4.5 kHz, which were similar to the two peaks at 1.5–2.5 and 5–7.5 kHz in rats reported by Bigelow et al [3] and Akache et al [13]. It seems that a third peak appears at 7 kHz, however, a measurement using higher frequencies (>8 kHz) is needed to clarify this peak in the future.

### Effects of perforation locations on the umbo vibration

**Figure 4:**
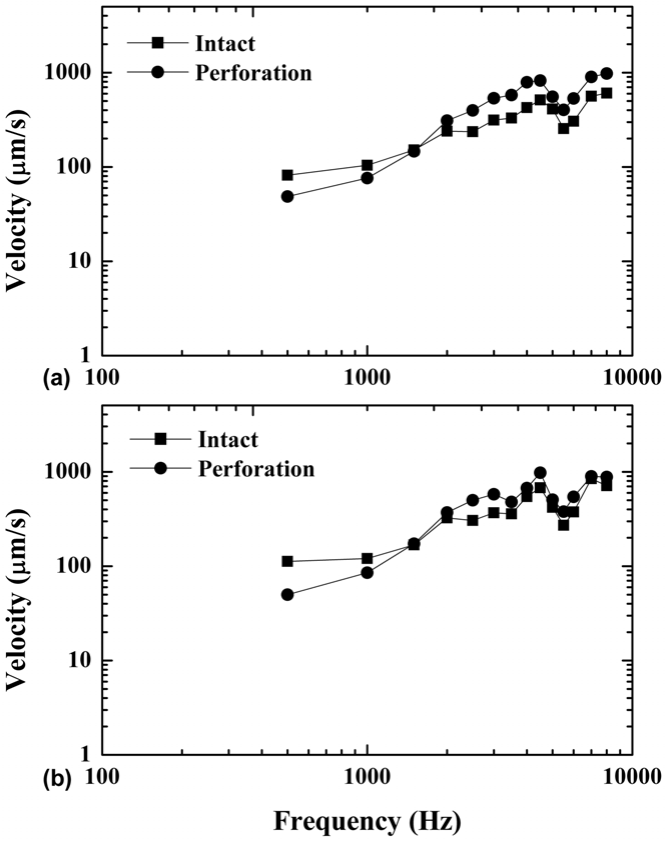
Mean velocities at the umbo from 12 bullas before and after perforation are presented. The upper and lower panels are mean velocities record from the perforations in the anterior-inferior quadrants of left ears (a) and in the posterior-inferior quadrants of right ears (b). Velocity losses were noticed below 1.5 kHz and negative losses at higher frequencies (2–8 kHz).

In the present investigation we compared the umbo velocity losses between perforations in the AI quadrant of left ears and in the PI quadrant of right ears in 6 guinea pigs. The shape of the perforations made by a needle appeared to be rhombic or fusiform. Mean velocities at the umbo from 12 bullas before and after perforations are shown in Fig. 4. The shape of the frequency response curves of the AI and PI perforations were very similar. Five to 7 dB velocity losses were noticed at 0.5 kHz in the PI perforated ears. However, the velocity losses became smaller at 1.0 kHz, approached 0 at 1.5 kHz, and became negative losses at higher frequencies (2–8 kHz). However, there are no statistical differences in velocity loss between AI and PI perforations at 0.5 kHz although the PI perforations may cause more velocity loss at low frequencies (paired *t*-test, *p*_0.5 kHz_ = 0.492, *p*_1 kHz_ = 0.904, *p*_1.5 kHz_ = 0.929).

### Comparison of the velocity responses recorded at different points on the manubrium

**Figure 5:**
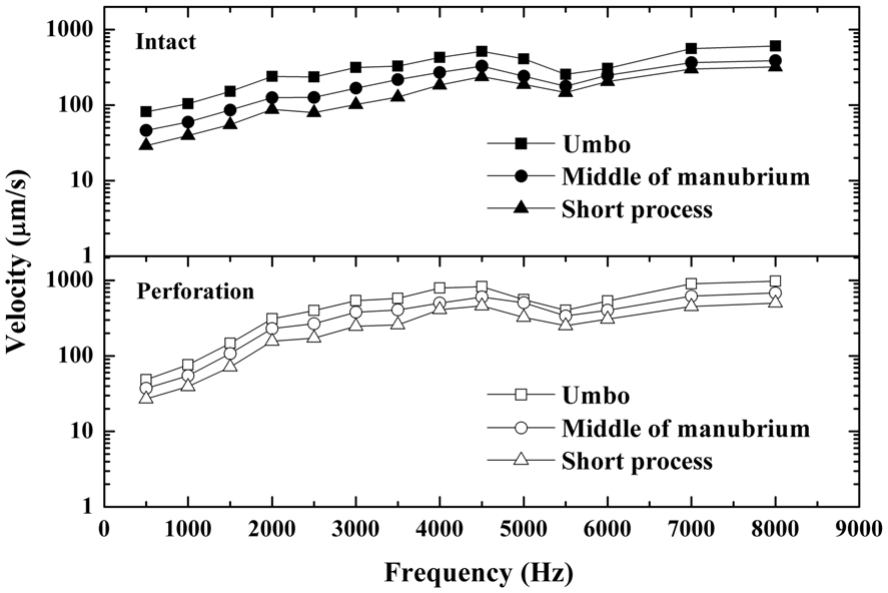
Average velocity responses recorded from 3 points on the manubrium of the intact TM (upper panel) and the AI quadrant perforated TM (lower panel). Shapes of the frequency responses were quite similar at all three points on the manubrium while the highest velocities were recorded at the umbo. The average ratio of the short process velocities to the umbo velocities was approximately 0.5 at all frequencies.

**Figure 6:**
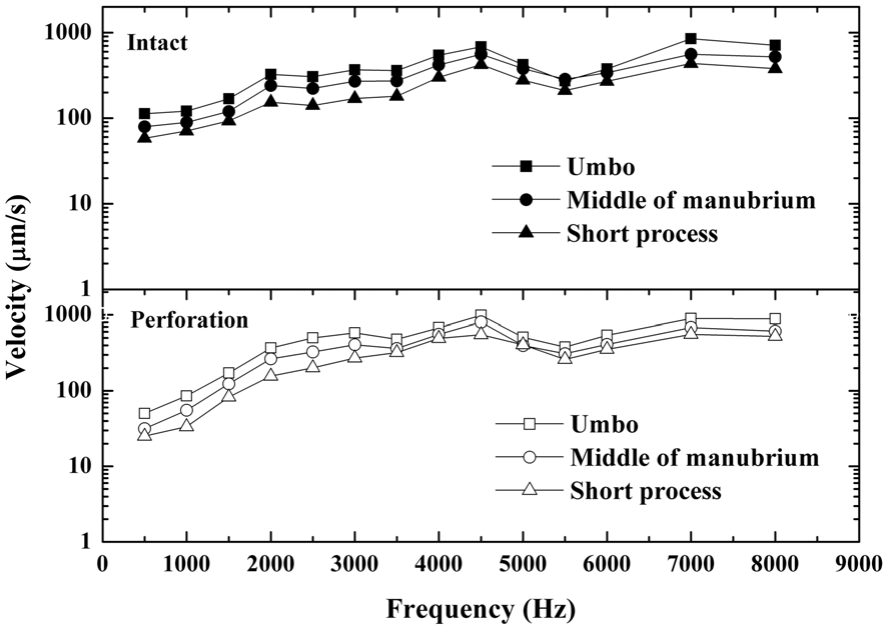
The average velocity responses recorded from 3 points on the manubrium of the intact TM (upper panel) and the PI quadrant perforated TM (lower panel). A similar pattern was found in the PI perforations compared to the AI perforations.

The velocity responses were measured at three points on the manubrium in 12 bullas before and after perforations made in AI or PI quadrants. The measurement locations are illustrated in Fig. 2 and the mean velocities for 3 points before and after perforations are shown in Figs. 5 and 6.

The curves in Figs. 5 and 6 displayed the manubrium velocities recorded at different points of the manubrium before (upper panels) and after (lower panels) perforations made in AI (Fig. 5) or PI (Fig. 6) quadrants. The highest velocities were recorded at the umbo while the lowest at the short process throughout the frequency range. However, the shapes of the frequency responses recorded at all three points on the manubrium were quite similar. The average ratio of the short process velocities to the umbo velocities was approximately 0.5 at all frequencies.

A similar pattern of the manubrium velocities was found in the perforated TM compared to the intact TM. There are no significant differences in the ratios of the short process velocities to the umbo velocities between the intact and the perforated TMs at all frequencies (*p*>0.05) except at 7 kHz (*p* = 0.004). These results confirm that the umbo is the most sensitive site to measure the manubrium vibration.

## Discussion

### Frequency dependence of velocity loss with perforations

The measurement of the umbo velocity in the perforated TMs demonstrated clearly that the manubrium velocity loss was frequency dependent in the present investigation: small TM perforations in the inferior quadrants caused small amount of velocity loss at 0.5 kHz and the velocity loss decreased toward zero as frequency increased toward 1.5 kHz. Because the shape of the perforations made by a 20-gauge needle was not round and covered less than 5% of the TM [14]. Transmission was reduced only by 0–7 dB, which was smaller than the results reported by Voss [9] and Gan [15]. A possible explanation is the perforation made by a needle in the present study was rhombic, which was smaller in size than a round perforation with a diameter of 1.1 mm. However, previous investigations have indicated that conductive hearing loss caused by tympanic membrane perforations was frequency-dependent: the largest hearing loss at low frequencies [3], [6], [10], [15]. Both present results and the reports mentioned above are consistent with the clinical observation that the TM perforations in human results in a low-frequency conductive loss. A possible interpretation is that the TM perforations exhibit a lower impedance magnitude to sound volume flow (into the tympanic cavity) at frequencies below 0.5 kHz, but an increasing impedance magnitude at higher frequencies [3].

### Effect of perforation locations on transmission

In order to determine whether the perforation locations play any role in the middle ear transfer function, we compared the changes of the umbo velocities from the perforations in the AI or PI quadrants of the TM. The results presented here showed no significant differences in the velocity losses between PI and AI perforations although there was a trend of velocity loss at low frequencies (below 1.5 kHz) for the PI perforations. This result is inconsistent with the generally accepted clinical opinion that a PI perforation would cause more hearing loss than an AI one at lower frequencies [7], [8]. In recent clinical investigations, Mehta et al [10] found that anterior perforations had smaller air-bone gaps than posterior perforations although there was no statistically significant difference. Ibekwe et al [11] also found that the location of the TM perforations had no effects on the degree of hearing loss in patients with acute TM perforations with various sizes. For patients with chronic otitis media, the PI perforation has more significant effects on the hearing loss than the AI one because the patients with PI perforation usually had impaired ossicular chain motion [7], [11]. However, larger perforations might result in more vibration velocity losses at lower frequencies. Therefore, the effects of different sites of larger perforations on middle ear sound transmission should be studied in the future.

### Effect of perforation on vibration pattern

Fleischer [16] hypothesized early that the mammalian middle ears have two rotation axes with two clearly defined modes of the malleus vibration: one axis is approximately parallel to the manubrium at low frequencies, and the other is approximately perpendicular to the manubrium at high frequencies. Saunders and Summers [17] investigated the auditory function of the middle ear by measuring vibrations at the short process and the umbo in the mouse, and their results have confirmed Fleischer's hypothesis. The results of the present study has showed that the ratio of short process velocity to the umbo velocity was approximately 0.5, and no systematic differences of the ratio were found before and after perforation (both AI and PI perforation) at any frequency. These data suggest that there is an axis perpendicular to the manubrium below 8 kHz in guinea pigs, which contradicts Fleischer's hypothesis. Supporting our results, Akache et al [13] observed a significantly low ratio in half of six rats. The TM of guinea pig, as rat, is much bigger than that of mouse, and size of the TM may changes the rotation axis modes. Therefore, each species may have its own middle ear rotation axes.

Our data has demonstrated that the manubrium velocity losses caused by TM perforations are frequency-dependent with the largest losses at low sound frequencies. However, manubrium velocity losses caused by small acute AI and PI perforations in guinea pigs have no significant impact on middle ear sound transmission at any frequency tested. The manubrium vibration axis may be perpendicular to the manubrium below 8 kHz in guinea pigs.
